# A coordination polymer for the site-specific integration of semiconducting sequences into DNA-based materials

**DOI:** 10.1038/s41467-017-00852-6

**Published:** 2017-09-28

**Authors:** Lamia L. G. Al-Mahamad, Osama El-Zubir, David G. Smith, Benjamin R. Horrocks, Andrew Houlton

**Affiliations:** 1 Chemical Nanoscience Labs, School of Chemistry, Newcastle University, Newcastle upon Tyne, NE1 7RU UK; 2 Department of Chemistry, College of Science, Al-Mustansiriya University, Baghdad, Iraq; 3 Present Address: School of Chemistry, University of Sydney, Sydney, NSW 2006 Australia

## Abstract

Advances in bottom-up material design have been significantly progressed through DNA-based approaches. However, the routine integration of semiconducting properties, particularly long-range electrical conduction, into the basic topological motif of DNA remains challenging. Here, we demonstrate this with a coordination polymer derived from 6-thioguanosine (6-TG-H), a sulfur-containing analog of a natural nucleoside. The complexation reaction with Au(I) ions spontaneously assembles luminescent one-dimensional helical chains, characterized as {Au^I^(μ-6-TG)}_*n*_, extending many μm in length that are structurally analogous to natural DNA. Uniquely, for such a material, this gold-thiolate can be transformed into a wire-like conducting form by oxidative doping. We also show that this self-assembly reaction is compatible with a 6-TG-modified DNA duplex and provides a straightforward method by which to integrate semiconducting sequences, site-specifically, into the framework of DNA materials, transforming their properties in a fundamental and technologically useful manner.

## Abstract (web summary)

Integration of semiconducting properties into the basic topological motif of DNA remains challenging. Here, the authors show a coordination polymer derived from 6-thioguanosine that complexes with Au(I) ions to form a wire-like material that can also integrate semiconducting sequences into the framework of DNA materials.

## Introduction

DNA, biology’s information carrier, is now an established part of preparative chemistry’s arsenal for materials synthesis^1–3^. Its robust nature, reliable synthesis, controllable length scale, allied with sequence coding for programmable self-assembly address many of the criteria desired of a materials design toolkit. The ability to incorporate pre-synthesized inorganic components, particularly nanoparticles, with oligonucleotides provides a logical approach to materials design. This has been demonstrated by the exquisite control over nanoparticle assembly to form DNA-based materials with tunable optical properties^4–6^, reconfigurable superlattice structures^7, 8^, and magnetic and catalytic properties^9–11^. However, the integration of semiconducting properties, particularly electrical conduction, into the basic topological framework of duplex DNA remains challenging.

Though studies on the electrical conductivity of duplex DNA have provided conflicting evidence^12–14^, numerous experiments now indicate that it is not an effective solid-state conductor beyond nm lengths^15–18^. Furthermore, effective charge transport appears to require physiological, solution-based, conditions (aqueous buffer etc.) to ensure retention of regular duplex conformation^19–22^. Conditions not well suited to solid-state device applications etc.

Initial indications that, so-called, metallo-DNA or M-DNA^23–25^ offered a solution to long-range electrical conductivity have not been widely adopted; possibly due to conflicting reports^26–29^ and the lack of selectivity over the location of metal-ion incorporation. Modified bases, termed ligandosides, capable of providing specific, non-natural, metal-ion-binding modes have been incorporated into duplex DNA as a means of introducing new properties^30–38^. However, while elegant these approaches are synthetically demanding, and, to date, none provide the extended, delocalized, bonding suited for effective electrical conductivity.

Simple thiopurines are sulfur-containing analogs of natural nucleosides, commonly used in treating a variety of medical conditions. They are readily incorporated into nucleic acids which, in fact, is a feature of their mode of action upon metabolism^39^. Their metal-ion specificity and binding modes are different from the natural derivatives, most notably in their aurophilicity and an ability to form coordination chain polymers via a single-atom-bridge as {−Metal−µS−}_*n*_^40, 41^. We hypothesized that targeting the synthesis of a gold-based polymer with such a nucleoside variant could provide a material with the required extended bonding motif. Importantly, the aurophilicity of the thiol group would avoid competing reactions with metal-ion-binding sites on the natural nucleosides and allow such a motif to be readily integrated into the framework of duplex DNA structures. Here, we show that the sulfur-containing analog of a natural nucleoside, 6-thioguanosine (6TG-H) reacts with gold(I) ions to spontaneously assemble luminescent helical chains that are structurally analogous to DNA. Uniquely, for such a material, this gold-thiolate coordination polymer can be transformed into a wire-like conducting form. We show that this self-assembly reaction is compatible with a 6-TG-modified DNA duplex and provides a straightforward method by which to integrate semiconductive domains, site-specifically, into DNA materials.

## Results

### Au(I)-thioguanosine preparation and characterization

The reaction of equimolar equivalents of Au(I) ions with the sulfur-modified nucleoside, 6-thioguanosine (6TG-H) in aqueous-based solution at neutral pH spontaneously forms a luminescent hydrogel (Supplementary Figs. 1, 2). The broad luminescence ~650 nm is characteristic of the formation of oligomeric gold-thiolate species^42, 43^. Further characterization by a range of techniques established the reaction to yield a gold(I)-thiolate coordination polymer of general formula {-Au-µ-6TG-}_*n*_ (Supplementary Discussion and Supplementary Figs. 1–11).

**Fig. 1:**
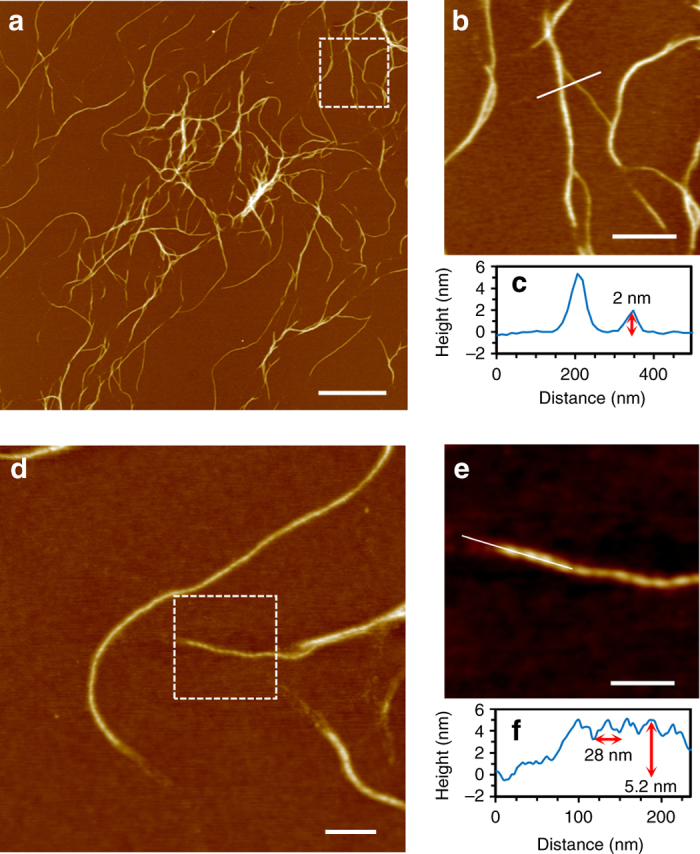
AFM images of Au-thioguanosine xerogel **1** drop-cast onto a silicon wafer. Large-scan (**a**) and a zoom area (**b**) of AFM height images of **1**. The associated cross-section (**c**) along the *white line* in **b**. AFM height images (**d**, **e**) showing the helical structure of individual molecular chains. The associated cross-section (**f**) along the *white line* in **e**. *Scale bar* is 2 µm in image **a**, 500 nm in image **b**, 200 nm in image **d**, and 100 nm in image **e**

Atomic force microscopy (AFM) imaging (Fig. 1) shows that the dried, xerogel, **1**, comprises one-dimensional molecular strands extending many microns in length with the smallest feature heights of ca. 2 nm (Fig. 1b). These strands entangle to form the necessary three-dimensional network required for gel formation, and can also be seen to intertwine to form larger diameter fibers (Fig. 1a–c). The AFM data reveals alternating light-dark features along individual fibers consistent with a highly organized helical structure (Fig. 1d, e)^42^. The height of these features indicates that they contain several individual strands and the apparent periodicity seen by AFM arises from intertwining in some relatively ordered supramolecular structure. In support of this, circular dichroism (CD) shows the appearance of markedly more intense bands across the UV-visible range compared to the 6TG-H alone (Supplementary Fig. 4). The longer wavelength bands at ca. 385 and 420 nm are attributed to metal-ligand charge transfer of the polymer backbone and confirm a helical nature to the main coordination chain.

X-ray diffraction (XRD) data from **1** did not feature sharp Bragg reflections, but instead showed broad peaks indicating that the sample was amorphous (Supplementary Discussion, Supplementary Figs. 5, 6, and Supplementary Table 1). The data were analyzed using a simplified Rietveld method by fitting a regression model comprising a sum of six Gaussian functions. The dominant peaks in Supplementary Fig. 5 are in the range 20 < *Q* < 40 and correspond to distances *d* = 2*π*/*Q* of 1.87, 2.31, and 3.18 Å. We assign these to C–S, Au–S, and Au…Au distances, respectively, and the larger 17.6 Å distance can be interpreted as the diameter of the helix.

**Fig. 2:**
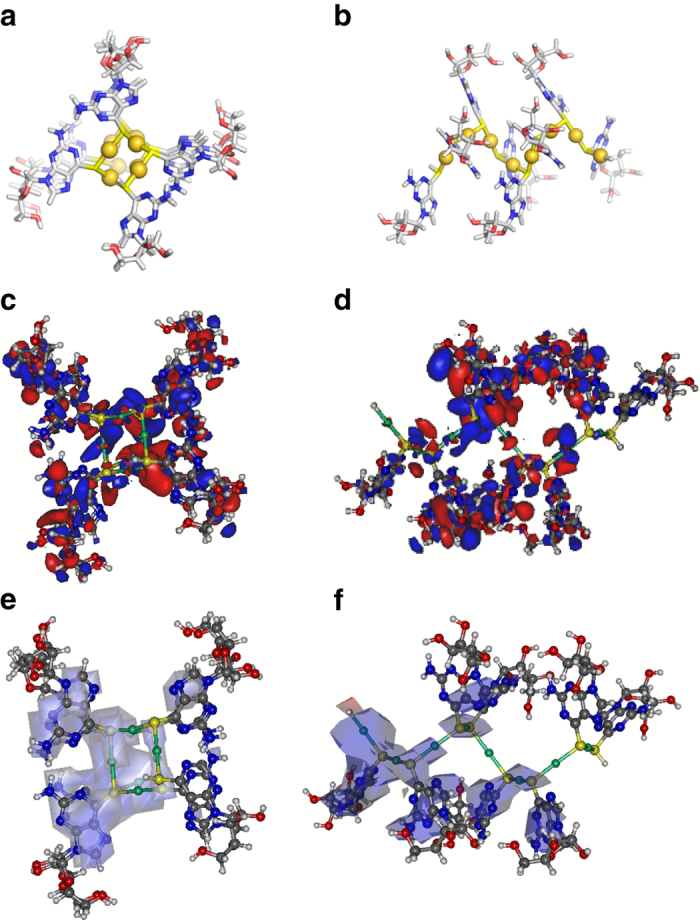
A model of the coordination polymer chain of {Au(I)-thioguanosine}_*n*_ viewed along and onto the helical chain axis. **a**, **b** Single point energy DFT calculations for an oligomer (Au_8_6TG_8_) using the SBKJC effective core potential and basis set with the B3LYP functional. The helical conformation shown is based on the geometry of comparable Au-thiolates determined by XRD. **c** View of the HOMO looking along the axis of the helix; **d** The HOMO from a direction normal to the helical axis; **e** Spin density map for the singly-charged radical cation (Au_8_6TG_8_)^+^. Looking along the helical axis and **f** the spin density map from a direction normal to the helical axis

The available data are all consistent with **1** having a helical structure based on thioguanosine bridging adjacent metal ions analogous to Au(I)-coordination polymers derived, for example, from thiomalate^44^. A molecular model of **1** based on this is shown in Fig. 2. The molecular structure has a central polymeric Au(I)-thiolate backbone around which the nucleobase and ribose groups extend. The resulting helical polymer is strikingly similar in size, topology, and surface functionality to duplex DNA.

### Electronic structure and electrical properties

**Fig. 3:**
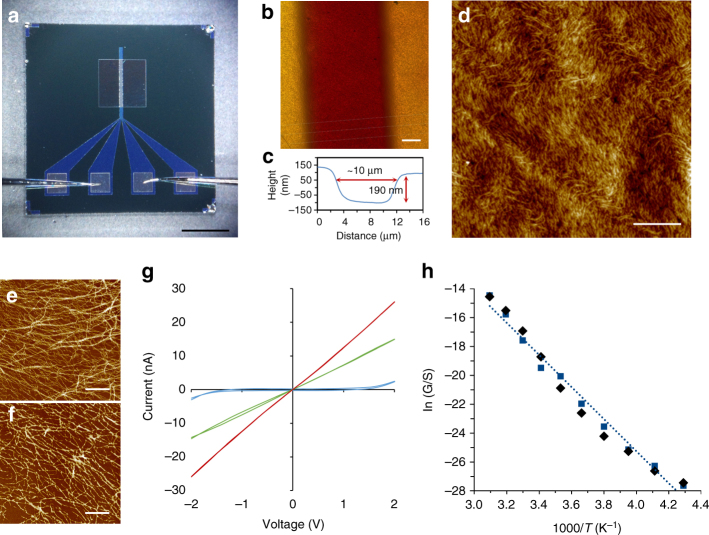
Electrical characterization of the Au-thioguanosine coordination polymer, **1**. **a** Patterned platinum MBE showing the contact pads at the *bottom* and the 10 µm spaced electrodes above. **b**, **d** AFM imaging of the electrode gap region. **c** A profile of the gap region indicating the size. AFM images before (**e**) and after (**f**) treatment of the xerogel with I_2_. **g** I-V curves of the **1** before (*blue*) and after doping with tris(4-bromophenyl)ammoniumyl hexachloroantimonate (*red*) and iodine (*green*). **h** An Arrhenius plot for the zero-bias conductance of the I_2_-doped Au-thioguanosine xerogel. Activation energy (*E*_a_) = 0.97 ± 0.02 eV obtained from two independent heating (*black*)/cooling (*blue*) cycles. *Scale bars* are 1 mm in image **a** and 2 µm in images **b**, **d**–**f**

Since the as-prepared material has both an extended structure and a long-wavelength visible luminescence (*λ*_max_ ~650 nm), we naturally considered if it exhibited useful semiconducting properties, analogous to those of conjugated organic polymers^45^. Density functional theory (DFT) calculations of the electronic structure show substantial delocalization of the HOMO, which includes major contributions from the {Au-µS}_*n*_, backbone as well as minor contributions from the π-system of the nucleobase. When a single electron is removed from the system and the spin density is computed at the same geometry, this also shows delocalization over both the {Au-µS}_*n*_ chain and the nucleobase (Fig. 2e, f). The changes in the mean atomic partial charges (Supplementary Table 1) are largest for the gold-thiolate backbone but with some contribution from the nucleobase. Together these findings suggested that oxidative doping might produce a conductive material and, indeed, this is case. Figure 3 shows data for the electrical characterization of **1** using a device constructed by depositing the gel between platinum microband electrodes (MBE) (Fig. 3a–d). After drying, this provided strands of **1** spanning the electrode gap. AFM imaging after treatment with I_2_ vapor showed that no notable structural changes occurred and the molecular strands remained intact (Fig. 3e, f). The chemical effects of this treatment, judged by X-ray photoelectron spectroscopy (XPS), indicated the reaction generally proceed via a redox mechanism with the formation of a polyiodide-doped coordination polymer, (**1**_**ox**_) (Supplementary Fig. 7 and Supplementary Discussion).

Current-voltage curves collected before (**1**) and after oxidation (**1**_**ox**_) (Fig. 3g) reveal a dramatic increase in conduction upon treatment. Essentially ohmic behavior is observed over the ±2 V range with an increase of at least two orders of magnitude in conductance upon treatment. The temperature-dependent I-V measurements show Arrhenius behavior characteristic of a thermally activated conduction mechanism with an activation energy of 94 kJ mol^−1^ for **1**_**ox**_ (Fig. 3h). Alternative oxidants such as (BrC_6_H_4_)_3_NSbCl_6_ produced similar behavior (Fig. 3g) confirming the conductive nature of the doped coordination polymer (**1**_**ox**_) and that the observed conductivity was not due simply to the presence of the polyiodide species^46^.

The data in Fig. 3 permit several conclusions regarding the nature of the conduction in **1**_ox_. First, it can clearly be seen that the IV curve shows little evidence of conductivity for the undoped material. The differential conductance at zero bias was estimated as <30 pS. The current at high bias, greater than about 1.5 V, shows some increase, but this shape of IV curve, which has almost zero slope at the origin, is not evidence of conductivity in the usual sense. Oxidative doping of **1** does produce a material with finite zero bias conductance, whether the oxidant employed is a vapor such as I_2_ (7.1 +/− 0.05 nS) or a liquid such as a dry acetonitrile solution containing (BrC_6_H_4_)_3_NSbCl_6_ (12.9 +/− 0.09 nS). Photoemission spectra of the doped and undoped material (Supplementary Fig. 7 and Supplementary Discussion) indicate that the positive charge introduced by oxidation resides mainly on Au and S atoms. This is supported by DFT calculations, which show large changes in atomic charge for Au and S, but much smaller changes for atoms of the nucleobase. We conclude that **1** shows electronic conductivity upon oxidation and the charge carriers are dominantly associated with the Au–S chains of the structure and not the nucleobase. The IV curves show ohmic behavior, i.e., the slope of the IV curve is the same no matter how small or large the deviation from equilibrium. This behavior is distinct from photoinduced electron transfer experiments in which long-range electron transfer is observed for photon energies sufficient to populate the initial excited state and distinct from electrochemical experiments where the applied potential must exceed the formal potential of the relevant couple to drive electron transfer.

Second, the data in Fig. 3h show that the zero bias conductance increases with temperature in accordance with an equation of Arrhenius form. This is characteristic of a semiconductor or hopping conductor (Supplementary Discussion) and not of a metallic conductor. In principle, the increase in conductance could be explained in terms of thermal excitation across a gap, which would result in an activation energy of ½*E*_gap_. However, the activation energy of about 0.97 eV does not match the data from optical spectroscopy. Fluorescence excitation spectra (Supplementary Fig. 3) show an optical gap of 480 nm, which is about 2.6 eV. Instead, the nature of the conductivity in doped **1**_**ox**_ is more like that of the conjugated polymers than crystalline inorganic semiconductors. Typically conjugated polymers are poorly conducting when undoped and have optical gaps of several eV, but conduct via a hopping mechanism when doped. Although bulk samples of conjugated polymers typically show temperature-dependent conductances which follow variable range hopping models^47^, we have observed simple Arrhenius behavior when they are constrained to form as one-dimensional structures^48^. In these systems, and **1**_**ox**_, the activation energy extracted from the Arrhenius plot is much smaller than the optical gap because it corresponds to thermally assisted tunneling between somewhat localized states rather than carrier generation by excitation across the gap as in crystalline inorganic semiconductors. Finally, it is worth noting that we observed essentially identical Arrhenius plots for both heating and cooling cycles and the doped polymer **1**_**ox**_ is stable up to at least 50 °C.

### Site-specific incorporation into DNA duplex

Next, we sought to establish the compatibility of this self-assembly chemistry with DNA oligonucleotides for the site-specific incorporation of this semiconducting Au^I^-thiolate motif into DNA sequences. To this end we designed an 18-mer oligonucleotide with the sequence 5′-SSSSACGCGAATTCGCGT-3′ (where S = 6-deoxythioguanosine, dTG-H), which contains a self-complementary 14-mer region. This was annealed to form the corresponding duplex {**OligoS**_**4**_} with 5′ overhangs with four consecutive 6-dTG-H groups. CD spectroscopy confirmed the formation of B-form duplex with negative–positive bands seen at ~250 and ~284 nm (Supplementary Fig. 9). Longer wavelength bands were also observed at ~330 nm (negative) and ~354 nm (positive) which are due to transitions associated with the 6-dTG-H sequence. Reactions of {**OligoS**_**4**_} with Au(I) ions exhibited an orange luminescence similar to **1** ca. 520 nm indicating the spontaneous formation of oligomeric Au(I)-thiolate species (Supplementary Fig. 10). The blue-shift in this emission compared to **1** can be rationalized by the short length of the Au-thiolate region in the **Au-{OligoS**_**4**_}. The self-assembly is also indicated by CD with a red-shift in the signal associated with the 6-dTG sequence and a transformation of the duplex region to an A-form conformation (Supplementary Fig. 9).

**Fig. 4:**
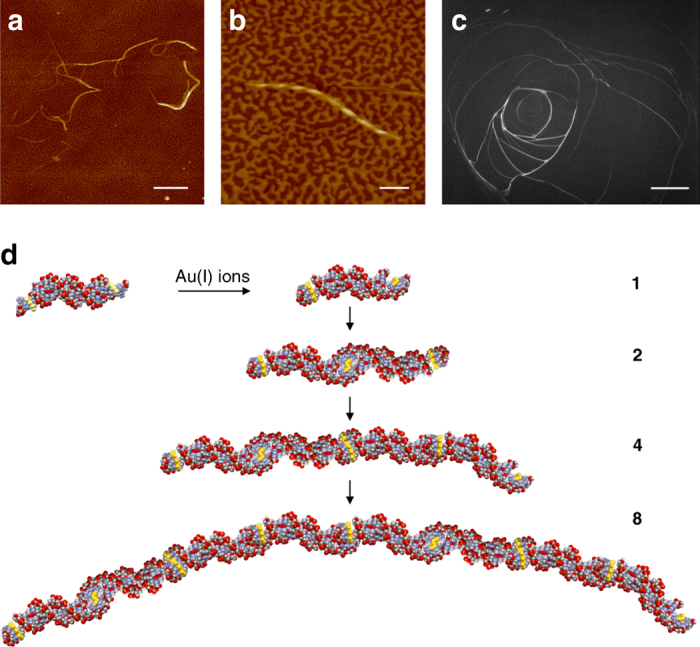
Au-thiolate-DNA concatemers. AFM height image of **Au-**[**OligoS**_**4**_] revealing the extended, micron, lengths of strands formed (**a**) and the helical nature of an individual structure (**b**). **c** Fluorescence image of **Au-**[**OligoS**_**4**_] revealing the macroscopic fibers. **d** Schematic illustration of the formation of DNA-semiconductor concatemer sequence strands by metal-ion-driven self-assembly. *Yellow* indicates the semiconducting Au-thiolate region, *red* = Oxygen, *purple* = Nitrogen, *gray* = Carbon. *Scale bar* is 500 nm in image **a**, 100 nm in image **b**, and 1 mm in image **c**

The successful integration and compatibility of the coordination polymer motif with the DNA duplex was revealed by AFM. This showed one-dimensional **Au-{OligoS**_**4**_} structures extending up to several microns in length indicating coaxial alignment of the native duplex and the gold-thiolate sequences (Fig. 4a, b). The observed one-dimensional structures suggest that branching while, in principle, possible is not dominant as this would form a cross-linked network. Branched structures may in principle arise from either two or more strands that intertwine for part of their length or by joining three duplexes via Au-S links. The former case can be distinguished in AFM by the diameter change at the branch point. Examination of the data (Fig. 4a and Supplementary Fig. 11a–f) shows that this is the typical case. Similar large features are not present in samples of {**OligoS**_**4**_} alone; these samples generally contain much shorter (100 s nm) fibers that can aggregate into bundles (Supplementary Fig. 12e–h). The mechanism by which the **Au-{OligoS**_**4**_} strands are produced is driven by coordinate bond formation involving the 6-thioguanosine terminal regions. While other donor atom sites (i.e., N, O) are present in the nucleoside bases, the high affinity of Au(I) for the mutant thiol-functionality provides the sequence-specificity for the coordination motif. The resulting hybrid material comprise alternating sections of native double helices linked by semiconducting oligo-Au(I)-TG regions (Fig. 4). As individual {**OligoS**_**4**_} are only ca. 6 nm long extensive concatenation involving hundreds and thousands of individual DNA duplexes occurs. In fact, fluorescence microscopy indicates that these assemblies can be macroscopic, extending in length to several mm (Fig. 4c). Closer inspection of individual structures of **Au-{OligoS**_**4**_} reveals, again, an alternating pattern of light-dark features along the length indicating helicity in the hybrid strands (Fig. 4b). The periodicity is consistent with the highly organized underlying structure expected from the self-assembly process outlined in Fig. 4d.

## Discussion

In summary, we have prepared a luminescent one-dimensional coordination polymer based on the self-assembly of Au^I^ ions with the sulfur-modified nucleoside, 6-thioguanosine. The material’s molecular structure is highly analogous to duplex DNA and this polymeric gold-thiolate, uniquely to our knowledge, exhibits electrical conduction upon oxidative doping. This semiconducting motif can be readily incorporated, co-axially and sequence-specifically, into the framework of DNA structures due to the metal-ion binding that assembles the coordination chain being specific for the mutant nucleoside. With the increasingly important role of DNA in bottom-up nanoscience and material synthesis^2, 4–6, 8, 9, 11^, routes to integrate technologically useful physico-chemical properties with DNA are highly sought. This has typically been achieved with pre-synthesized components such as nanoparticles, or by deposition of metals and inorganics on the DNA^49^. Our work advances the field of DNA materials toward further complexity by allowing the incorporation of semiconducting properties via an alternative method and makes possible new types of construction protocols, compositional architectures, and material combinations.

## Methods

### Materials

Reagents were obtained from Sigma-Aldrich. NANOpure® deionized water (18 MΩ cm resistivity) was obtained from a NANOpure® DIamond™ Life Science ultrapure water system equipped with a DIamond™ RO Reverse Osmosis System (Barnstead International). The thio-modified 18-mer oligonucleotide, 5′-SSSSACGCGAATTCGCGT-3*′* where S = 6-thiodeoxyguanosine, containing a 14-mer self-complementary region was obtained from ATDBio (Southampton, UK). Oligonucleotides were made up in 10 mM HEPES buffer containing 50 mM NaCl and adjusted to pH = 7.

### Preparation of **1**

Sample-spanning gels of **1** were prepared as follows. An aqueous suspension of 6-thioguanosine (6TG-H, 10 mg) was sonicated for 30 s to achieve a fine dispersion and to this was added an equimolar equivalent of Au(I) ions, formed by reduction of HAuCl_4_ with two equivalents of thiodiglycol, HO(CH_2_)_2_S(CH_2_)OH in a final volume of 600 μl. Upon addition of the Au(I) ion a yellow-orange gel is formed which is stable to inversion in a vial (Supplementary Fig. 1). Alternatively, reactions could be performed in a (4:1) H_2_O/MeOH mixture with dissolution of the 6TG-H by adding NaOH. The remainder of the reaction was performed as above. These gels contain chloride ions from the gold salt and the thiodiglycol-derived oxidation product as the corresponding sulfone, HO(CH_2_)_2_S=O(CH_2_)OH, as indicated by XPS (see below) and confirmed by single-crystal X-ray crystallography in the latter case.

### FTIR spectroscopy

FTIR spectra (in the range 800–4000 cm^−1^) were recorded in transmission mode on a Varian 800 Scimitar Series. Samples of **1** (~2 µl) was deposited on a clean p-Si(100) chip (1 cm^2^) and dried in air for 1 h prior to analysis (Supplementary Fig. 3).

### Electrospray mass spectrometry

ES-MS spectra were recorded with a Waters Micromass LCT Premier mass spectrometer. Sample requirements for this analysis necessitated dissolution of **1** in methanol:water (50:50) with gentle heating.

### Fluorescence spectroscopy

Emission and excitation spectra were recorded on a SPEX Fluoromax spectrofluorimeter. Gels of **1** are luminescent, exhibiting an emission band at ~650 nm (*λ*_ex_ = 480 nm) (Supplementary Fig. 2).

### Circular dichroism

CD spectra were recorded on a Jasco J-810 spectrometer. CD spectra of aqueous 6-thioguanosine (1 mg ml^−1^) were recorded in a quartz cell with pathlength 0.2 mm. CD spectra of **1** as a gel (prepared with 10 mg/1.8 ml of 6-thioguanosine) were recorded in a quartz cuvette of pathlength 0.1 mm. In order to facilitate direct, quantitative comparison of the two samples, the ellipticity for the 6-thioguanosine (Supplementary Fig. 4a) has been multiplied by a factor 10/3.6 to account for the difference in concentration and pathlength.

### Atomic force microscopy

AFM data was acquired using a Multimode 8 atomic force microscope with a NanoscopeV controller (Bruker) and a “E” scanner. Nanoscope software version 9.1 was used to control the microscope. The system was operated in ScanAsyst in Air mode as a peak force tapping mode at ultra-low forces minimize damage to the samples. For reducing vibrational noise, an isolation table/acoustic enclosure was used (Veeco Inc., Metrology Group). Silicon tips on silicon nitride cantilevers (ScanAsyst, Bruker) were used for imaging. The nominal tip radius was approximately 2 nm, resonant frequency 150 kHz, and spring constant *k* ~0.7 nm^−1^. The AFM data were analyzed with NanoScope Analysis 1.5 software (Bruker). The sample was prepared by adding 2 µl of **1** onto a clean silicon wafer and drying in air.

### Powder XRD

XRD data were acquired using of a PANalytical X’Pert Pro diffractometer (PANalytical) using Cu Kα radiation source (*λ* = 1.540 Å). Samples of **1** were dried onto glass slides.

### X-ray photoelectron spectroscopy

XPS spectra were acquired using a Thermo Scientific K-Alpha X-ray photoelectron spectrometer (Thermo Electron Corp., East Grinstead, UK), equipped with an Al Ka X-ray source (1486.6 eV). A take-off angle of 90° was used during data acquisition, and a charge neutralization gun used to compensate for surface charging. The CasaXPs software (Casa, http://www.casaxps.com, UK) was used to analyze the XPS spectra. All spectra were corrected to hydrocarbon C1s peak at 285 eV as reference. Wide scans spectra were recorded at a pass energy of 150 and 1 eV/step, while narrow scans spectra were recorded at a pass energy of 50 and 0.1 eV/step.

### Electrical measurements

All of the electrical measurements were carried out under dry nitrogen without light illumination. Current-voltage measurements at different temperatures were measured on the probe station (Cascade Microtech with a B1500A parameter analyzer, Agilent) using a thermal chuck system (Model ETC-200L, ESPEC, Japan). Platinum MBEs (Smart Microsystems Pt MB-4000, Windsor Scientific Ltd., Slough, UK) were used to fabricate electronic devices for electrical characterization of Au-thioguanosine xerogel. The MBEs were made on Si/SiO_2_ substrates. Four independent platinum electrodes were patterned on the top of the SiO_2_ layer. The height of the electrodes is 200 nm and their width is 10 µm with 10 µm spaces between them. The surfaces of the MBEs were electrically insulated except for a 2 × 2 mm^2^ area for depositing the gel, as shown in Fig. 3a. Devices were fabricated by depositing the gel onto the platinum MBEs. The platinum MBEs were washed with ethanol and dried with nitrogen gas. The clean platinum electrodes were analyzed on a probe station and reference current/voltage curves were recorded, which showed the background currents to be less than 100 fA at 2 V. For the device a drop (~1 µl) of Au-thioguanosine gel **1** was cast onto the uninsulated area on the MBE. The device was left to dry for a week in Schlenk flask, which was maintained on a vacuum line (~750 mbar) at a temperature of 45 °C. The gel droplet dried to produce a film of the Au-thioguanosine fibers across the Pt-electrodes, as shown in Fig. 3b, d. The electrodes were connected to the probe station and current/voltage curves were collected. For doping, the xerogel **1** was exposed to iodine or tris(4-bromophenyl)ammoniumylhexachloroantimonate, (BrC_6_H_4_)_3_NSbCl_6_. In the former case, **1** on platinum MBEs was exposed to iodine vapor for 1 h by heating 0.1 g of iodine to 45 °C in a 50 ml round bottom glass flask. For the latter **1** on MBEs was treated with a drop (~1 µl) of 0.1 mol dm^−3^ of (BrC_6_H_4_)_3_NSbCl_6_ in anhydrous acetonitrile. The gel was left to dry for 30 min before the current/voltage curves were collected. Control experiments where iodine or (BrC_6_H_4_)_3_NSbCl_6_ was applied to MBEs in the absence of **1** showed negligible background currents. In other control experiments DNA was deposited on MBEs and subsequently treated in a similar manner to the samples for **1** (Supplementary Fig. 8).

### Reaction of 6-TG-modified oligonucleotides with Au(I) ions

An 18-mer oligonucleotide, **OligoS**_**4**_, containing a 14-mer self-complementary sequence [5′-SSSSACGCGAATTCGCGT-3′ where S = 6-thiodeoxyguanosine], with a tetrameric 5′-6-thiodeoxyguanosine overhang region was annealed by heating to 70 °C and cooled to room temperature. Duplex formation was confirmed by CD, which indicated the expected negative-positive bands seen at ~250 and ~284 nm for B-form DNA^50^. Longer wavelength bands were also observed at ~330 nm (negative) and ~354 nm (positive) which are due to transitions associated with the 6-thioguanosine section (Supplementary Fig. 9)^51^.

A typical reaction of the [**OligoS**_**4**_]-duplex and Au(I) ions was as follows: to 20 µl of a 1 mM [**OligoS**_**4**_] solution was added an Au(I) solution (prepared by reduction of HAuCl_4_.3H_2_O using two equivalents of thiodiglycol) to give an gold:sulfur ratio of 1:1. This mixture exhibited an orange luminesce (Supplementary Fig. 10a–c) and a very broad emission peak at ~520 nm (Supplementary Fig. 10d). The CD spectra of **Au-**[**OligoS**_**4**_] shows bands at ~275 nm (positive), ~340 nm (negative), and 375 nm (positive). These changes indicate a transformation to a more A-like conformation of the native duplex region as well as changes to the thioguanosine section^50^.

### Fluorescence microscopy

Fluorescence images were obtained using a Zeiss Epifluorescence Microscope equipped with a Hg lamp and a bandpass filter for the excitation of 300–400 nm. A long-pass filter with a cut off at 420 nm was used for detection and the image was collected with a monochrome CCD camera.

### Data availability

All data are available from the authors upon reasonable request.

## Electronic supplementary material

Supplementary Information

## References

[1] Meng WJ (2016). An autonomous molecular assembler for programmable chemical synthesis. Nat. Chem..

[2] Jones MR, Seeman NC, Mirkin CA (2015). Programmable materials and the nature of the DNA bond. Science.

[3] Aldaye FA, Palmer AL, Sleiman HF (2008). Assembling materials with DNA as the guide. Science.

[4] Sun D, Gang O (2011). Binary heterogenous superlattices assembled from quantum dots and gold nanoparticles with DNA. J. Am. Chem. Soc..

[5] Young KL (2014). Using DNA to design plasmonic metamaterials with tunable optical properties. Adv. Mater..

[6] Park DJ (2015). Plasmonic photonic crystals realized through DNA-programmable assembly. Proc. Natl. Acad. Sci. USA.

[7] Kim Y, Macfarlane RJ, Mirkin CA (2013). Dynamically interchangeable nanoparticle superlattices through the use of nucleic acid-based allosteric effectors. J. Am. Chem. Soc..

[8] Macfarlane RJ, Jones MR, Lee B, Auyeung E, Mirkin CA (2013). Topotactic interconversion of nanoparticle superlattices. Science.

[9] Zhang C (2013). A general approach to DNA-programmable atom equivalents. Nat. Mater..

[10] Radha B (2014). Reconstitutable nanoparticle superlattices. Nano Lett..

[11] Auyeung E (2015). Controlling structure and porosity in catalytic nanoparticle superlattices with DNA. J. Am. Chem. Soc..

[12] Fink HW, Schonenberger C (1999). Electrical conduction through DNA molecules. Nature.

[13] Dekker C, Ratner MA, Singh RRP (2001). Electronic properties of DNA. Phys. World.

[14] Endres RG, Cox DL, Singh RRP (2004). The quest for high-conductance DNA. Rev. Mod. Phys..

[15] Storm AJ, van Noort J, de Vries S, Dekker C (2001). Insulating behavior for DNA molecules between nanoelectrodes at the 100 nm length scale. Appl. Phys. Lett..

[16] Gomez-Navarro C (2002). Contactless experiments on individual DNA molecules show no evidence for molecular wire behavior. Proc. Natl. Aacd. Sci. USA.

[17] Bockrath M (2002). Scanned conductance microscopy of carbon nanotubes and lambda-DNA. Nano Lett..

[18] Sonmezoglu S, Sonmezoglu OA, Cankaya G, Yildirim A, Serin N (2010). Electrical characteristics of DNA-based metal-insulator-semiconductor structures. J. Appl. Phys..

[19] Guo XF, Gorodetsky AA, Hone J, Barton JK, Nuckolls C (2008). Conductivity of a single DNA duplex bridging a carbon nanotube gap. Nat. Nanotechnol..

[20] Slinker JD, Muren NB, Renfrew SE, Barton JK (2011). DNA charge transport over 34 nm. Nat. Chem..

[21] Sontz PA, Muren NB, Barton JK (2012). DNA charge transport for sensing and signaling. Acc. Chem. Res..

[22] O’Brien E (2017). The 4Fe4S cluster of human DNA primase functions as a redox switch using DNA charge transport. Science.

[23] Aich P (1999). M-DNA: a complex between divalent metal ions and DNA which behaves as a molecular wire. J. Mol. Biol..

[24] Rakitin A (2001). Metallic conduction through engineered DNA: DNA nanoelectronic building blocks. Phys. Rev. Lett..

[25] Whittell GR, Manners I (2007). Metallopolymers: new multifunctional materials. Adv. Mater..

[26] Moreno-Herrero F (2003). Topographic characterization and electrostatic response of M-DNA studied by atomic force microscopy. Nanotechnology.

[27] Liu B, Bard AJ, Li C-Z, Kraatz H-B (2005). Scanning electrochemical microscopy. 51. Studies of self-assembled monolayers of DNA in the absence and presence of metal ions. J. Phys. Chem. B.

[28] Mizoguchi K, Tanaka S, Ogawa T, Shiobara N, Sakamoto H (2005). Magnetic study of the electronic states of B-DNA and M-DNA doped with metal ions. Phys. Rev. B.

[29] Spring BQ, Clegg RM (2007). Fluorescence measurements of duplex DNA oligomers under conditions conducive for forming M−DNA (a metal−DNA complex). J. Phys. Chem. B.

[30] Clever GH, Kaul C, Carell T (2007). DNA-metal base pairs. Angew. Chem. Int. Ed..

[31] Clever GH, Shionoya M (2010). Metal-base pairing in DNA. Coord. Chem. Rev..

[32] Takezawa Y, Shionoya M (2012). Metal-mediated DNA base pairing: alternatives to hydrogen-bonded Watson-Crick base pairs. Acc. Chem. Res..

[33] Atwell S, Meggers E, Spraggon G, Schultz PG (2001). Structure of a copper-mediated base pair in DNA. J. Am. Chem. Soc..

[34] Wagenknecht HA (2003). Metal-mediated DNA base pairing and metal arrays in artificial DNA: towards new nanodevices. Angew. Chem. Int. Ed..

[35] Sinha I, Guerra CF, Mueller J (2015). A highly stabilizing silver(I)-mediated base pair in parallel-stranded DNA. Angew. Chem. Int. Ed..

[36] Johannsen S, Megger N, Bohme D, Sigel RKO, Muller J (2010). Solution structure of a DNA double helix with consecutive metal-mediated base pairs. Nat. Chem..

[37] Scharf P, Muller J (2013). Nucleic acids with metal-mediated base pairs and their applications. ChemPlusChem.

[38] Santamaría-Díaz N, Méndez-Arriaga JM, Salas JM, Galindo MA (2016). Highly stable double-stranded DNA containing sequential silver(I)-mediated 7-deazaadenine/thymine Watson–Crick base pairs. Angew. Chem. Int. Ed..

[39] Aarbakke J, Janka-Schaub G, Elion GB (1997). Thiopurine biology and pharmacology. Trends Pharmacol. Sci..

[40] Olea D (2005). From coordination polymer macrocrystals to nanometric individual chains. Adv. Mater..

[41] Amo-Ochoa P (2013). Coordination chemistry of 6‑thioguanine derivatives with cobalt: toward formation of electrical conductive one-dimensional coordination polymers. Inorg. Chem..

[42] Lavenn C (2015). A luminescent double helical gold(I)–thiophenolate coordination polymer obtained by hydrothermal synthesis or by thermal solid-state amorphous-to-crystalline isomerization. J. Mater. Chem. C.

[43] Odriozola I, Loinaz I, Pomposo JA, Grande HJ (2007). Gold-glutoathione supramolecular hydrogels. J. Mater. Chem..

[44] Bau R (1998). Crystal structure of the antiarthritic drug gold thiomalate (myochrysine): a double-helical geometry in the solid state. J. Am. Chem. Soc..

[45] Skotheim, T. A. & Reynolds, J. *Handbook of Conducting Polymers, 2 Volume Set*. 1680 (CRC Press, 2007).

[46] Hardy LC, Shiver DF (1986). Poly (ethylene oxide)-sodium polyiodide conductors: characterization, electrical conductivity, and photoresponse. J. Am. Chem. Soc..

[47] Joo J (2000). Physical characterization of electrochemically and chemically synthesized polypyrroles. Macromolecules.

[48] Hassanien R (2010). Preparation and characterization of conductive and photoluminescent DNA-templated polyindole nanowires. ACS Nano.

[49] Houlton A, Watson SMD (2011). DNA-based nanowires. Towards bottom-up nanoscale electornics. Ann. Rep. Prog. Chem. A.

[50] Nordén, B., Rodger, A. & Dafforn, T. *Linear Dichroism and Circular Dichroism: A Textbook on Polarized-Light Spectroscopy* (RSC, 2010).

[51] Repges R, Beuck C, Weinhold E, Raabe G, Fleischhauer J (2008). 6-Thioguanosine in DNA as CD-spectroscopic probe to study local structural changes upon protein binding. Chirality.

