# Supplementary material for: A coordination polymer for the site-specific integration of semiconducting sequences into DNA-based materials
Source: Nat Commun. 2017 Sep 28;8:720. doi: 10.1038/s41467-017-00852-6 (PMC5620084; doi:10.1038/s41467-017-00852-6)
Supplement: Supplementary file 1 — Supplementary Information [file 41467_2017_852_MOESM1_ESM.pdf]

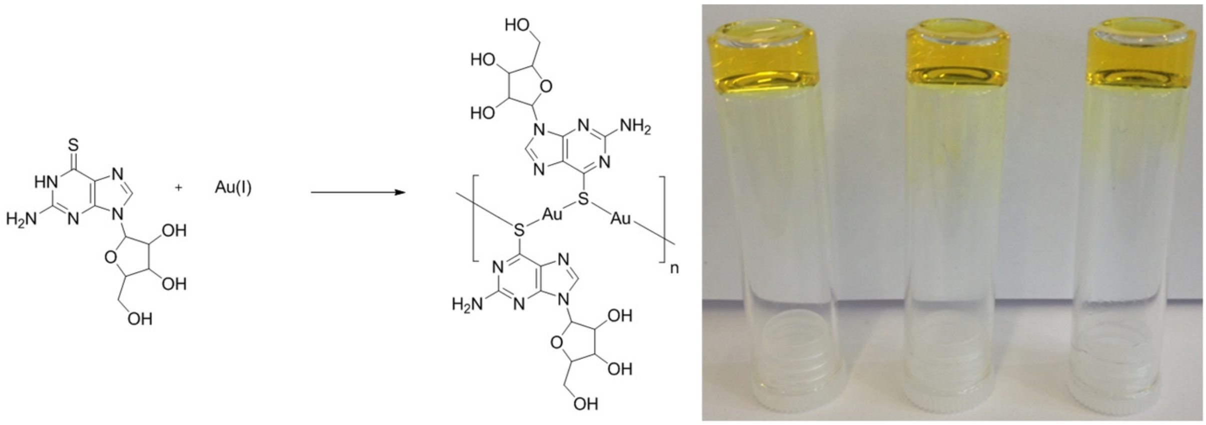

Supplementary Figure 1 | Reaction scheme for forming 1. Optical image of the Au-thioguanosine gel preparations, **1**, in inverted glass vials. The gel shown is *approx.* 96% solvent by mass.

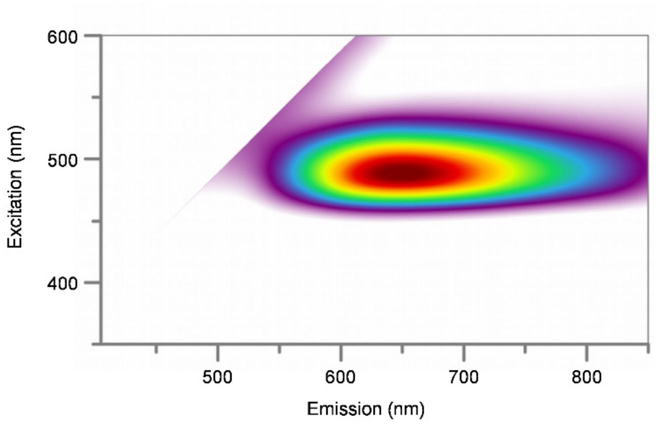

Supplementary Figure 2 | Normalised emission profile of gel 1 (λ_{ex}=480 nm).

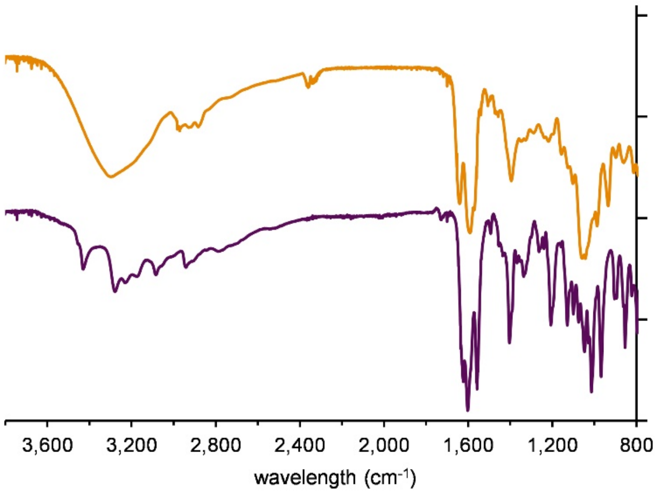

Supplementary Figure 3 | FTIR spectra of thioguanosine (bottom, purple) and 1 (top, orange).

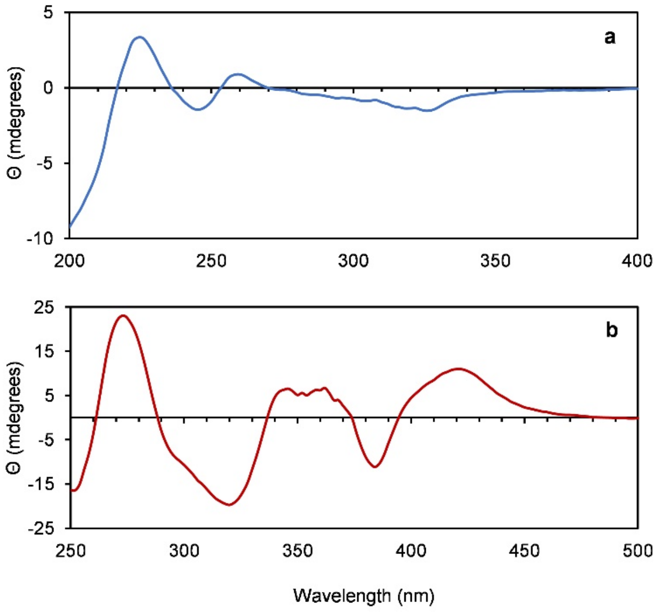

Supplementary Figure 4 | CD spectra of (a) 6-thioguanosine and (b) the Au(I)-thioguanosine gel, 1. For (**a**) the longer wavelength bands at *ca*. 385 nm and *ca*. 420 nm are attributed to metal-ligand charge transfer of the polymer backbone and confirm a helical nature to the main coordination chain.

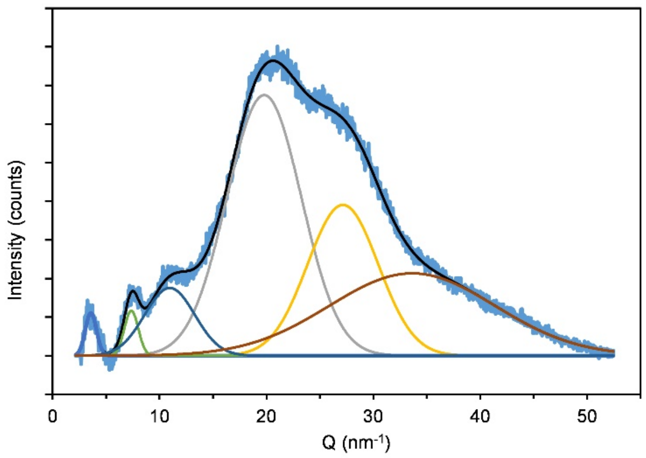

Supplementary Figure 5 | X-ray diffraction pattern for 1 after subtraction of the low angle (Porod) scattering) and a linear background. The fitted regression model (black line) was a sum of 6 Gaussian functions and the coloured lines shown the individual Gaussians.

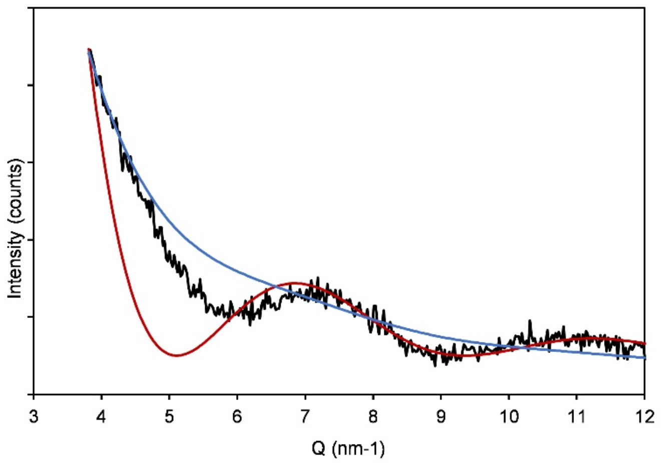

Supplementary Figure 6 | Low angle X-ray scattering data for 1. Experiment (black), the least squares fit of a regression model corresponding to scattering from a cylinder at normal incidence to the cylinder axis (red), and another fit for scattering from a cylinder, but averaged over orientation (blue).

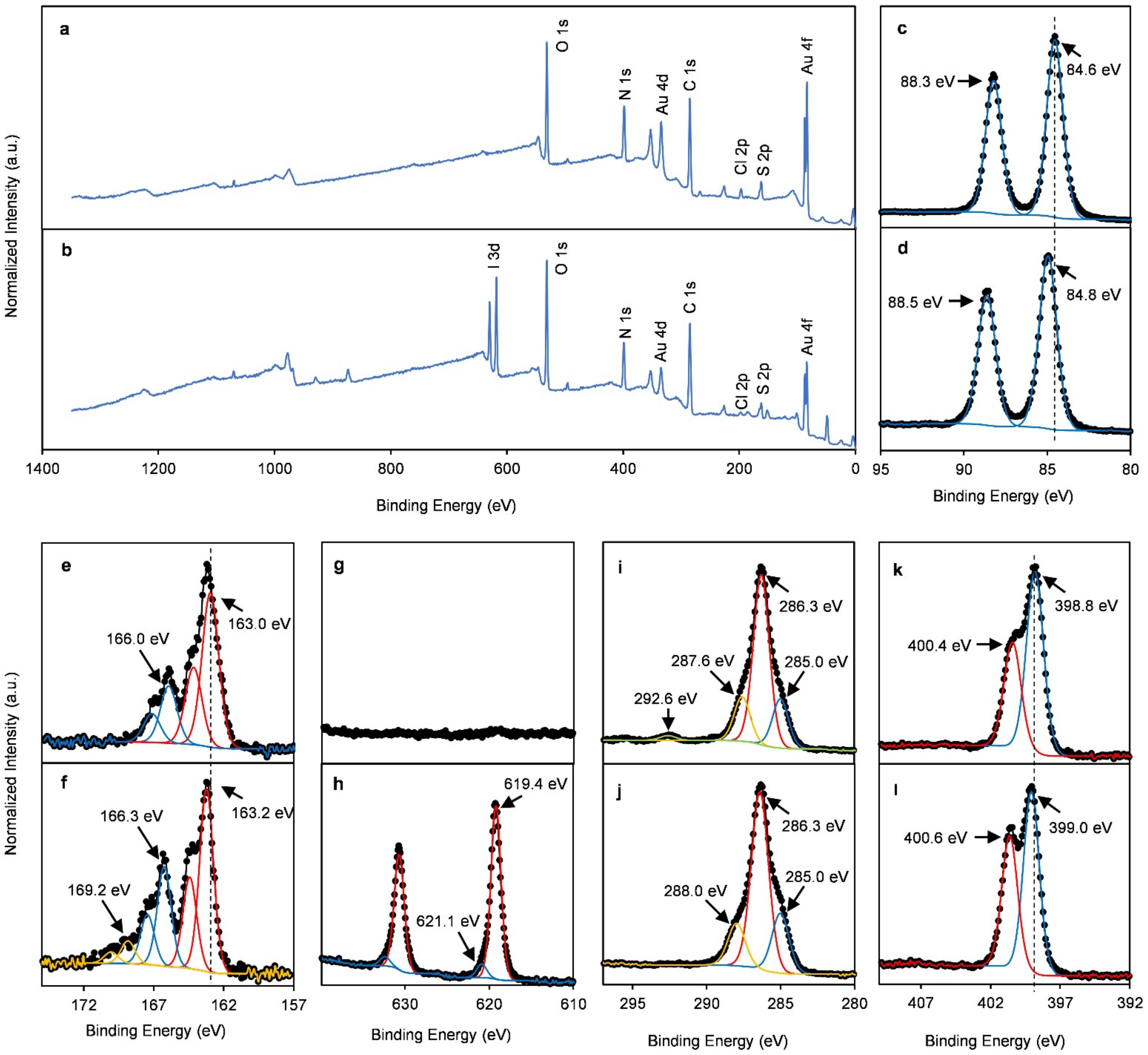

Supplementary Figure 7 | XPS spectra of (dried) Au-thioguanosine gel samples. XPS survey spectra of Au-thioguanosine gel samples (**a**) before and (**b**) after oxidation with iodine. High-resolution XPS spectra of Au 4f, S 2p and I 3d. (**c** and **d**) Au $4f_{5/2}$ and $4f_{7/2}$, (**e** and **f**) S $2p_{1/2}$ and $2p_{3/2}$, (**g** and **h**) I $3d_{3/2}$ and $3d_{5/2}$, (**i** and **j**) C 1s and (**k** and **l**) N 1s regions of the XPS spectra of Au-thioguanosine gel samples (**c**, **e**, **g**, **i** and **k**) before and (**d**, **f**, **h**, **j** and **l**) after doping with iodine.

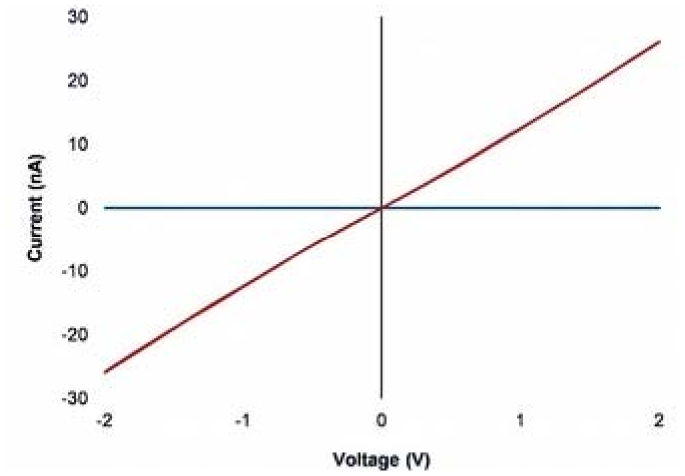

Supplementary Figure 8 | Electrical characterisation of Lambda DNA and the Au-thioguanosine gel. I-V curves of the λ-DNA before (blue) and after doping with Tris(4-bromophenyl)ammoniumyl hexachloroantimonate ($[BrC_6H_4)_3N]SbCl_6$) (green) and Au-thioguanosine coordination polymer after doping with ($[BrC_6H_4)_3N]SbCl_6$) (red). The blue and green curves for λ-DNA before and after treatment are indistinguishable from the x-axis. These are negative controls demonstrating the lack of conductivity in these samples.

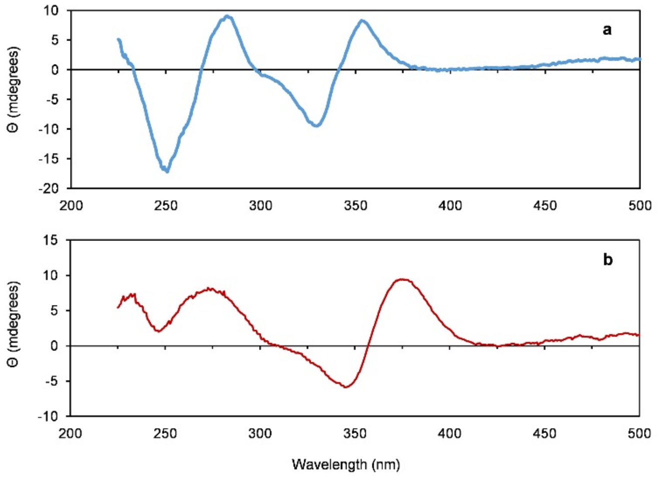

Supplementary Figure 9 | CD spectra for [OligoS_4] before and after reaction with Au(I) ions. CD spectra for (a) [Oligo**S**$_4$] and (b) **Au-**[OligoS_4].

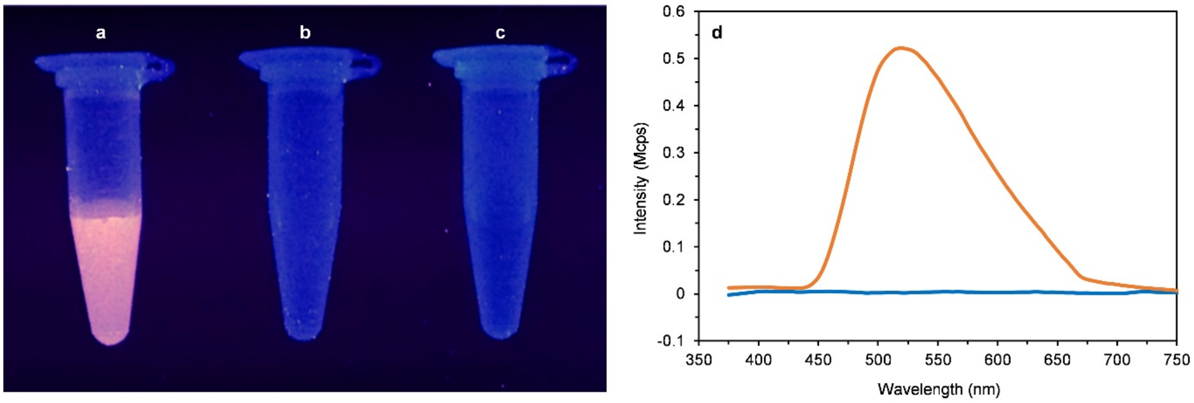

Supplementary Figure 10 | Optical image and Emission spectrum of Au-[OligoS$_4$] and controls. Optical image under illumination by an Hg lamp of (**a**) a solution of the **Au-[OligoS$_4$]**, (**b**) a solution of [OligoS$_4$] alone and (**c**) a solution of Au(I) ions. **d**, The emission spectrum of the OligoS$_4$ (blue) and Au-[OligoS$_4$] (orange. The excitation wavelength was 360 nm. The pathlength was 1 cm.

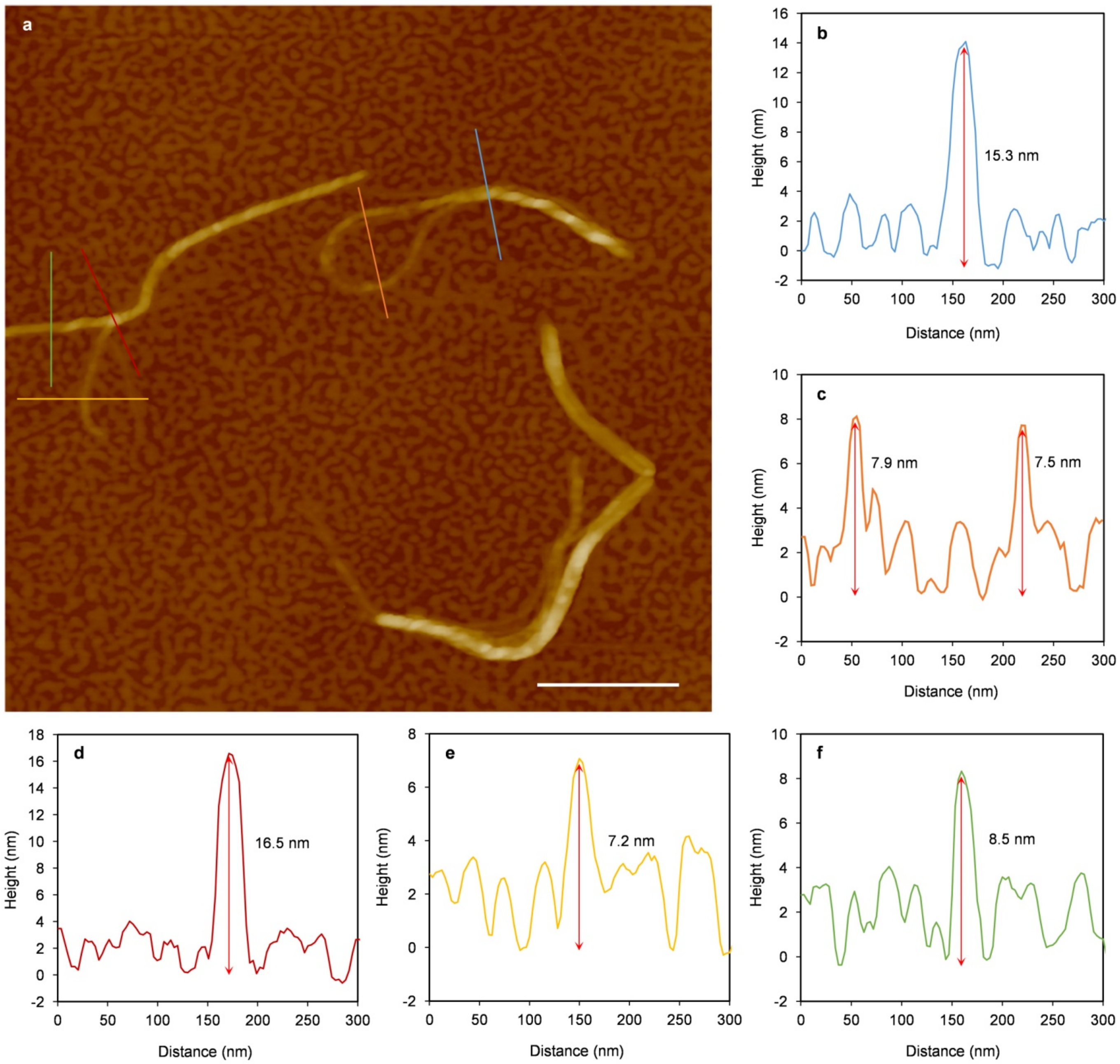

Supplementary Figure 11 | AFM topographical images of Au-[OligoS4]. a AFM height image of Au-[OligoS4] after deposition on a clean Si chip. AFM height image illustrating branching by intertwining of multiple strands of Au-[OligoS4] polymer. **b-f**, The associated cross-sections along the coloured lines in the image (**a**). Scale bar is 200 nm in the image (**a**).

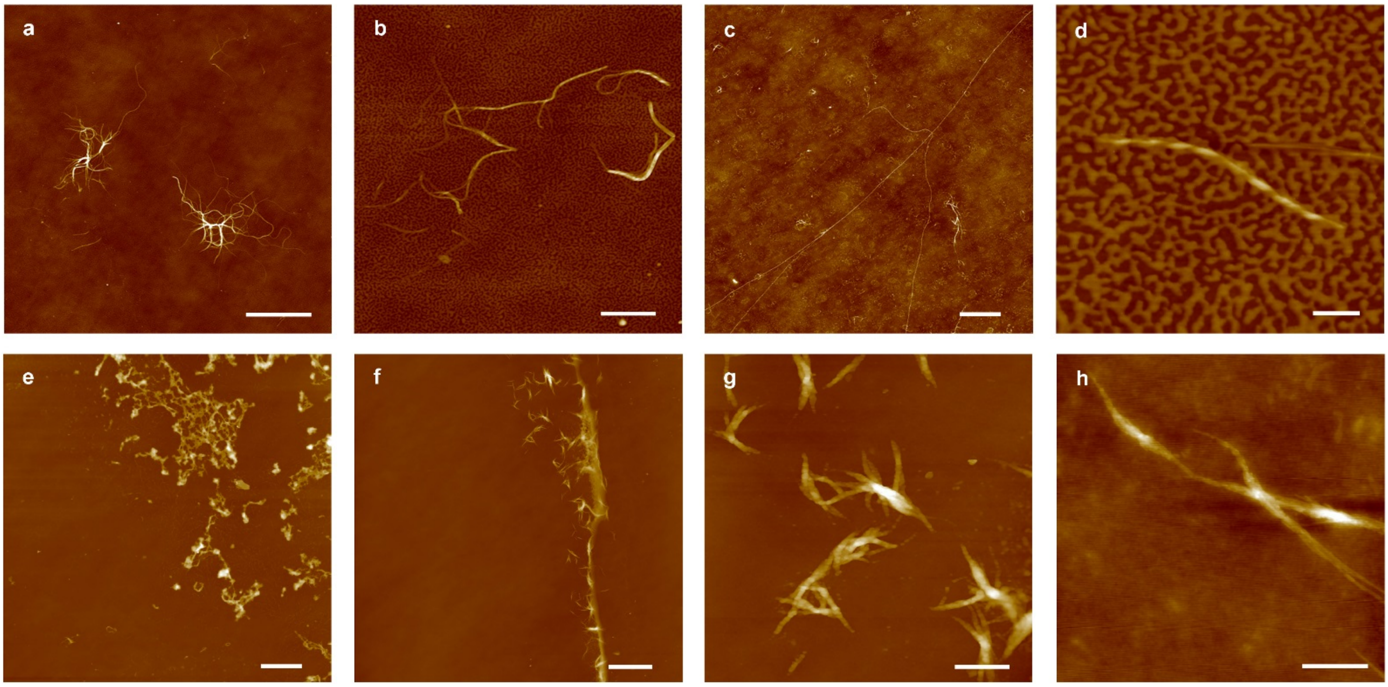

Supplementary Figure 12 | AFM topographical images of Au-[OligoS_4] and [OligoS_4] alone. a-d, AFM height image of Au-[OligoS_4] after deposition on a clean Si chip. A zoomed out (**a**) and zoomed in (**b**) region in AFM topographical image. **c**, AFM height image highlighting the extended, multiple micron, lengths of the Au-[OligoS_4] strands. The length of the diagonal strand in this image is more than 21 µm. **d**, AFM height image illustrating the helical nature of an individual strand. **e-h**, AFM images of [OligoS_4] alone**.** Typical structural features are < 800 nm, though some larger features are found due to aggregation. Scale bars are 2 µm in images (**a**, **c**, **e** and **f**), 500 nm in images (**b** and **g**), 100 nm in image (**d**) and 200 nm in image (**h**).

Supplementary Table 1 | Calculated changes in atomic charges (Mulliken & Löwdin) upon one-electron oxidation of ($Au_8$6TG_8).

Atom	Mulliken (e)	Löwdin (e)
Au	0.0254	0.0289
S	0.0114	0.0129
C	0.0029	0.0027
N	0.0017	0.0055
H	0.0039	0.0020
O	0.0008	0.0011

The values were obtained from single point energy DFT calculations using the SBKJC effective core potential and basis set with the B3LYP functional. The atomic charges are averaged over all the atoms of the same type in the molecule and are given in units of *e*.

Supplementary Discussion

FTIR spectroscopy. The FTIR spectrum of 6-TGH itself shows an intense *v*(C=S) stretch at 1205 cm^{-1} and the lack of absorbance corresponding to *v*(S-H), around 2600 cm^{-1}, indicating the parent compound is in the thione-, rather than thiol-, tautomeric form.[1,2] Upon reaction with Au(I) ions this band disappears indicating metal ion binding at the S6 group in the thiol-form, consistent with literature for Au(I)-6-thiopurine derivatives (Supplementary Fig. 2).[3-6]

Electrospray Mass Spectrometery. Electrospray mass spectrometry data of samples of **1** confirmed the presence of oligomers consistent with the formation of a coordination polymer. At low Au(I):ligand ratio (0.25-0.5 equivalents of Au(I) *per* 6-TG) positive-ion mode indicated the presence of the following species; $[Au(6\text{-}TGH)_2]^+$ (*m/z* (100%) Calc 795.10; Found 795.10), $[Au(6\text{-}TGH)_3]^+$ (*m/z* (100%) Calc 1094.17; Found 1094.17), $[Au_2(6\text{-}TGH)_2(6TG)]^+$ (*m/z* (100%) Calc 1290.13; Found 1290.13), $[Au_2(6\text{-}TGH)_3(tGs)]^+$ (*m/z* (100%) Calc 1589.20; Found 1589.23). At equimolar Au:6-TGs ratio a far greater number of species was observed containing multiple numbers of gold ions and 6-TGs sub-units. These included; $[Au_2(6\text{-}TGH)6TG]^+$ (*m/z* (100%) Calc 991.06; Found 991.0641); $[Au_4(6\text{-}TG)_3]^+$ (*m/z* (100%) Calc 1682.05; Found 1682.0957); $[Au_6(6\text{-}TG)_4]^{2+}$ (*m/z* (100%) Calc 1187.02; Found 1187.0178); $[Au_7(6\text{-}TG)_5]^{2+}$ (*m/z* (100%) Calc 1434.53, Found 1434.5452); $[Au_9(6\text{-}TG)_7]^{2+}$ (*m/z* (100%) Calc 1929.56; Found 1929.6716).

Fluorescence spectroscopy. Gels of **1** are luminescent, exhibiting an emission band at ~650 nm (λ_{ex} = 480 nm) Supplementary Fig. 3. This feature is consistent with the formation of coordination polymers, such as Au-thiophenolate and Au-glutathione[7,8] and Au-thiolate oligomers.[9,10] 6-Thioguanosine has an intrinsic luminescence with an emission at ~400 nm (λ_{ex} = 365 nm).

Circular dichroism. The CD spectra for 6-TG-H and the gel material **1** are shown in Supplementary Fig. 4. The former shows only weak bands in the UV-vis region due to the

chirality being derived from the ribose group which is not a strong chromophore in this region. In contrast **1** shows markedly more intense absorptions bands across the full spectral range. This indicates a highly organized self-assembly of the thioguanosine groups upon metal ion binding with the formation of a chiral, helical, arrangement. The appearance of a bands >350 nm, well into the visible region and attributed to the Au-thiolate charge-transfer, is strong evidence for the formation of a 1D-coordination polymer that is helical.

Atomic Force Microscopy (AFM). Fig. 1 shows AFM data for the dried, xerogel, **1** revealing the 1-D molecular strands spanning many microns in length. The smallest heights, corresponding to individual polymer strands, are approx. 2 nm. The alternating light-dark features along individual strands are consistent with a helical arrangement. Individual strands are seen to intertwine and entangle as is necessary for gelation.

Powder X-ray Diffraction (XRD). The X-ray diffraction data from **1** did not feature sharp Bragg reflections, but instead showed broad peaks indicating that the sample was amorphous, Supplementary Fig. 5 and 6. After removing the low-angle scattering (AQ^{-4}) and a linear background, the data was analysed using a simplified Rietveld method by fitting a regression model comprising a sum of 6 Gaussian functions. The dominant peaks in Supplementary Fig. 5 are in the range $20 < Q < 40$ and correspond to distances $d=2\pi/Q$ of 1.87, 2.31 and 3.18 Å. We assign these to C-S, Au-S and Au...Au distances respectively. Larger d values at 5.74, 8.54 and 17.6 Å are also apparent in the range $Q < 15$ shown in Supplementary Fig. 5. The 17.6 Å distance can be interpreted as the diameter of the helix, however this low angle scattering is modelled more directly in Supplementary Fig. 6.

Supplementary Fig. 6 shows a least squares fit of the low angle data to a cylinder model. (https://www.ncnr.nist.gov/resources/sansmodels/Cylinder.html; Guinier, A. and G. Fournet, "Small-Angle Scattering of X-Rays", John Wiley and Sons, New York, (1955)). The optimized value of the cylinder diameter is in the range 15 – 16 Å depending on whether the

scattering is averaged uniformly over all angles of incidence. The observed oscillations suggest some partial ordering of **1** on the substrate. The best-fit diameter is less than that observed in AFM, but consistent with the proposed structure of **1** in which the electron density is concentrated in the Au-S chain in the interior of the helix.

Molecular modeling and DFT calculations. A model for **1** consistent with the above data is shown in Fig. 2 as a $\{Au_8(\mu S\text{-}6TG)_8\}$ oligomeric chain. This is based on the single-crystal X-ray diffraction study of the analogous gold(I)-thiolate, derived from thiomalate.[11] This structural motif has also recently been observed for gold(I)-thiophenolate.[7] The model of **1** is a helical 1-D coordination polymer with a central $\{\text{-}\mu S6\text{-}Au\text{-}\}_n$ chain, about which the nucleosides are pendant. The featured μ2-S6 bridging mode is known for 6-thiopurine derivatives in forming both discrete[12,13] and polymeric coordination complexes.[14-16] The diameter of the helix in **1** at *approx*. 2.0 nm, as measured between hydroxyl-group oxygen atoms, is consistent with AFM data.

The DFT calculations used Firefly QC package[17], which is partially based on the GAMESS (US) source code,[18] with the SBKJC effective core potentials.[19,20]

X-ray photoelectron spectroscopy (XPS). Survey scans of xerogel samples of **1** identifying the elemental composition are shown in Supplementary Fig. 7c-h for before and after oxidative treatment with iodine; the latter was used to effect oxidative doping for electrical conduction. Along with the gelating product **1**, elements derived from starting materials are also observed. The survey spectra show the expected elemental composition of **1** (i.e. C, N, O, S and Au) along with Cl 2p at a binding energy (BE) of ~198 eV, attributed to chloride ions from the starting material ($HAuCl_4.3H_2O$). The I 3d peak appears in the survey spectrum after the oxidation treatment, at a BE of 618 eV.

Supplementary Fig. 7c and d shows the high-resolution spectra of Au $4f_{5/2}$ and $4f_{7/2}$ for **1**. Au $4f_{5/2}$ and $4f_{7/2}$ peaks are separated by 3.67 eV and the ratio of the component band areas is 6:8.

Prior to treatment with iodine the component at 84.6 eV is attributed to Au $4f_{7/2}$ and that at 88.3 eV to Au $4f_{5/2}$.[21,22] The BE value of the $4f_{7/2}$ peak indicates the +1 formal oxidation state for the Au ions[23] (typical range 84.3-85.5 eV).[24,25] After doping with iodine, both the Au $4f_{5/2}$ and $4f_{7/2}$ peaks are shifted to higher binding energy by 0.2 eV.

The high resolution XPS spectra for sulfur were fitted with two spin-orbit components ($2p_{1/2}$ and $2p_{3/2}$) with a fixed splitting ~1.2 eV and with the ratio between the band areas fixed at 1:2. The S $2p_{3/2}$ at BE 163 eV is attributed to sulfur in Au(I)-thiolate form.[22] This value, in fact, suggests a bridging thiolate mode in **1** based on previous reports which show higher S $2p_{3/2}$ binding energy (~163 eV)[26,27] for this compared to comparable terminal thiol binding (~162 eV)[28]. The S $2p_{3/2}$ peak at 166 eV is attributed to sulfoxide[29,30] (R-SO-R) formed by oxidation of the thiodiglycol, used to reduce Au(III) in the $HAuCl_4.3H_2O$ starting material to Au(I) ions. After iodine doping (Supplementary Fig. 7f), a new S $2p_{3/2}$ peak appears at a BE of 169.2 eV that is attributed to sulfone[22,30] (R-SO_2-R). From the calculation of the area of S $2p_{3/2}$ peaks before oxidation by iodine, on average 68.1 % of the sulfur in the samples is thiolate and the rest is sulfoxide. After the oxidation, both of these components are reduced with 63.8 % thiolate, 27.3 % sulfoxide and 8.8 % sulfone. These values show that only small fraction (~7 %) of the thiolate in the Au-thioguanosine has been oxidized to the corresponding sulfoxide- and sulfone-Au(I) complexes after treatment with iodine.[31] Additionally, the S $2p_{3/2}$ spectra is shifted after oxidation to a higher BE by 0.2-0.3 eV. The high resolution scan of XPS spectra for iodine were fitted with two spin-orbit components ($3d_{3/2}$ and $3d_{5/2}$) separated by 11.5 eV and with a component band area ratio of 2:3.[22] The I $3d_{5/2}$ spectrum of **1** was fitted with two components (619.4 and 621.1 eV), as shown in Supplementary Fig. 7h. The peak at 619.4 eV is attributed to I_3^-,[32,33] while the other peak at 621.1 eV is in the range of both I_2 [32,33] and I_5^- [33-36] as the binding energies of both are within a small range and can overlap. The presence of the I_5^- is possible in the presence of I_2 and I_3^- (I_2

+ $I_3^- \rightleftharpoons I_5^-$). The iodine doping process is expected to initially form I_3^-, which reacts subsequently through a reversible reaction with further I_2 to form relatively stable species of polyiodide.[35,36]

Since the XPS spectra do not show Au(III) it indicates that the Au-thioguanosine xerogel is doped by injecting charge into **1** forming polyiodide as counter anions rather than oxidative-addition of the I_2 to the metal centers.[37]

Supplementary Fig. 7i-l shows high resolution C 1s and N 1s spectra of **1** xerogel before and after doping. The C 1s spectra were fitted with four components. The first peak at 285 eV is attributed to contributions from both C–C and C=C.[38] The peak at 286.3 eV is attributed to C–O and C–N[22,38] and the peak at 287.6 eV is attributed to carbon atoms in N=C–N.[39] The peak at binding energy 292.6 eV is attributed to π–π^* shake up satellite peak.[38,40,41] This peak disappears after doping, Supplementary Fig. 7i and j. The N 1s spectra were fitted with two peaks at BE of 400.4 and 398.8 eV that are assigned to conjugated sp^2 (C=N) and non-conjugated sp^3 (C–N< and –NH_2) nitrogen atoms, respectively.[42]

The high resolution XPS spectra of the xerogel **1** show little observable change in the chemical composition, except the noted oxidation of 7 % of thiolate, after iodine treatment. The binding energy shift in the Au 4f, S 2p, and N 1s spectra by 0.2 eV compared to C 1s after treatment is indicates of oxidative doping of the coordination polymer **1**. In addition, the loss of the shake up satellite peak in the C 1s spectrum after doping is consistent with our DFT calculations of the change in electronic configuration upon oxidation.[43]

Reaction of 6-thiodeoxyguanosine-modified oligonucleotides with Au(I) ions. Structural analysis by AFM of **Au-**[OligoS_4] reactions showed the formation of long one-dimensional structures extending up to several microns in length (Supplementary Fig. 11a-d). Similar features are not prevalent in control samples of [OligoS_4] alone (Supplementary Fig. 11e-h). Since the length of an individual [OligoS_4] is *approx.* 6 nm (based on a 22-mer length

comprising a 14-mer duplex and 2 x 4-nucleotide overhangs) this indicates extensive catenation through metal-ion binding which cross-links hundreds of individual oligonucleotide duplex into extended structures. Upon closer inspection evidence of helicity is apparent from the alternating light-dark pattern along individual structures. Fluorescence microscope images of samples of **Au-**[OligoS_4] indicate that these assemblies can aggregate into macroscopic fibers (Fig. 4c).

Electrical characterization using microband electrodes. The experimental device used to measure the conductance of the Au-6TG polymer comprises Pt microbands of width 10 μm separated by gaps of 10 μm. The polymer is cast as a film across the device and forms contacts at each Pt microband. The device is of the form M/S/M, i.e., two metal/semiconductor interfaces back-to-back. This situation is distinct from a Schottky diode, which is a single M/S interface with an ohmic contact to the semiconductor. The I-V characteristic of an M/S interface is:

$$i = i_0\left(exp\left(\frac{eV}{kT}\right) - 1\right) \quad (1)$$

In order to keep the notation simple, the effect of the Schottky barrier at the interface is included in the potential V appearing in supplementary equation (1) by a translation of the potential scale.

The M/S/M device corresponds to two such diodes, with opposite bias to each other and an ohmic resistance for the material S inbetween, R_{bulk}. For small values of the potential, the characteristic of the diode linearises and it behaves as a resistor with (differential) conductance g:

$$g = i_0\frac{e}{kT} \quad (2)$$

The M/S/M system is symmetric and therefore the overall resistance is:

$$R = \frac{2kT}{ei_0} + R_{bulk} \quad (3)$$

As long as $R_{bulk} \gg \frac{2kT}{ei_0}$, then most of the applied bias is dropped across the bulk of S and the I-V characteristic is ohmic irrespective of whether S is a metal or semiconductor. It is not easy to distinguish semiconducting from metallic behavior of the film based on the I-V characteristic alone. However, the temperature dependence of the measured resistance R allows us to make the distinction because metallic conductors show an increased resistance as the temperature rises due to increased electron-phonon scattering rates and semiconductor crystals or polymers show a decreased resistance because of the effect of thermal activation on either carrier density or hopping rates (Fig. 3h, main text).

At sufficiently high bias, it may be possible to observe deviations from ohmic behavior (see undoped polymer in Figure 3g of the manuscript and discussion below). We and others have seen such effects in other polymer systems,[44-46] but we do not observe them in the present work for the *doped* polymer which shows ohmic behaviour consistent with supplementary equation (3). However, it can be seen that in the undoped polymer, potentials > 1.5 V produce a deviation from linearity, although the trace is still reversible on the return scan. This part of the I-V curve is similar to those observed for hopping polymers under conditions where the ionic countercharges are immobile.[44] It has a similar functional form to the Butler-Volmer equation.

$$I = \frac{n_p n F A \delta k_{ex}}{2D}\left[e^{-\rho\alpha\frac{\delta\ eV}{Lk_BT}} - e^{-\rho(1-\alpha)\frac{\delta\ eV}{Lk_BT}}\right] \qquad (4)$$

n and n_p are the densities of empty and occupied sites (by carriers), δ is the site-site hopping distance, L is the interelectrode spacing and k_{ex} is the electron self-exchange rate constant for site–site hopping at zero bias. D is the dimensionality of the system, α is a symmetry factor (about 0.5 in Marcus theory), and ρ is a non-ideality factor that is often interpreted in terms of clustering of sites. In the case of the undoped Au-6TG polymer n_p is very small and there is little current until charge is injected at the electrodes. It is worth noting that supplementary

equation (4) predicts the linear region at low bias and an Arrhenius-like temperature dependence.

Electrochemical effects and the DNA bases. We considered whether the I-V measurements could be interpreted in terms of electrochemical oxidation of the bases. However, the linearity and lack of hysteresis of the I-V data in Fig. 3g of the manuscript strongly argues against this. The lack of hysteresis (doped or undoped polymer) and the linear I-V characteristic (doped polymer) are inconsistent with an electrochemical process driven by the interfacial potential and especially with the electrochemistry of the DNA bases where chemical steps following the electron transfer prevent observation of the reverse process except at scan rates (10 - 100 V s^{-1}) orders of magnitude greater than applied in our work.[47] Instead, the lack of hysteresis is a piece of evidence in favour of our interpretation of the measurements in terms of electron transport in the film.

Supplementary References

1 Singh, K., Yadav, R. A. & Yadav, J. S. Vibrational studies of biomolecules—III. 6-thioguanine. *Spectrochim. Acta A Mol. Biomol. Spectrosc.* **47**, 819-820 (1991).

2 Kasende, O. E. Infrared spectra of 6-thioguanine tautomers. An experimental and theoretical approach. *Spectrochim Acta A* **58**, 1793-1808 (2002).

3 Cookson, P. D. & Tiekink, E. R. T. Crystal and molecular structures of tri(o-tolyl)phosphinegold(I) purine-6-thiolate ethanol solvate (1/1) and tri(c-hexyl)phosphinegold(I) 6-methyl-2-thiouracilate. *J. Chem. Crystallogr.* **24**, 805-810 (1994).

4 Cookson, P. D., Tiekink, E. R. T. & Whitehouse, M. W. Phosphinegold(I) complexes containing the purine-6-thiolate anion, and their antiarthritic activity. *Aust. J. Chem.* **47**, 577-586 (1994).

5 Ho, S. Y. & Tiekink, E. R. T. (6-Mercaptopurinato)(tricyclohexylphosphine)gold(I) ethanol solvate. *Acta. Crystallogr. Sect. E Struct. Rep. Online* **62**, M2855-M2857 (2006).

6 Vincent, B. R., Clarke, D. J., Smyth, D. R., de Vos, D. & Tiekink, E. R. Cytotoxicity of triorganophosphinegold(I) complexes of thiobenzoate. *Met. Based Drugs* **8**, 79-84 (2001).

7 Lavenn, C. *et al.* A luminescent double helical gold(I)–thiophenolate coordination polymer obtained by hydrothermal synthesis or by thermal solid-state amorphous-to-crystalline isomerization. *J. Mater. Chem. C* **3**, 4115-4125 (2015).

8 Odriozola, I., Loinaz, I., Pomposo, J. A. & Grande, H. J. Gold-glutoathione supramolecular hydrogels. *J. Mater. Chem.* **17**, 4843-4845 (2007).

9 Luo, Z. T. *et al.* From aggregation-induced emission of Au(I)-thiolate complexes to ultrabright Au(0)@Au(I)-thiolate core-shell nanoclusters. *J. Am. Chem. Soc.* **134**, 16662-16670 (2012).

10 Yu, Y. *et al.* Identification of a highly luminescent Au-22(SG)(18) nanocluster. *J. Am. Chem. Soc.* **136**, 1246-1249 (2014).

11 Bau, R. Crystal structure of the antiarthritic drug gold thiomalate (Myochrysine): a double-helical geometry in the solid state. *J. Am. Chem. Soc.* **120**, 9380-9381 (1998).

12 Pope, L., Laing, M., Caira, M. R. & Nassimbeni, L. R. Space group reported for dimeric 6-mercaptopurine copper(I) chloride complex. *Acta Crystallogr., Sect. B: Struct. Sci.* **32**, 612-613 (1976).

13 Shoemaker, A. L., Singh, P. & Hodgson, D. J. Structure of bis[dichloro-6-mercaptopuriniumcopper(I)]dihydrate - refinement. *Acta Crystallogr., Sect. B: Struct. Sci.* **32**, 979-980 (1976).
14 Dubler, E. & Gyr, E. New metal complexes of the antitumor drug 6-mercaptopurine. Syntheses and X-ray structural characterizations of dichloro(6-mercaptopurinium)copper(I), dichlorotetrakis(6-mercaptopurine)cadmium(11), and bis (6- mer captopur ina t 0) cadmium (11) dihydrate. *Inorg. Chem.* **27**, 1466-1473 (1988).
15 Amo-Ochoa, P. *et al.* Synthesis of designed conductive one-dimensional coordination polymers of Ni(II) with 6-mercaptopurine and 6-thioguanine. *Inorg. Chem.* **48**, 7931-7936 (2009).
16 Amo-Ochoa, P. *et al.* Assembling of dimeric entities of Cd(II) with 6-mercaptopurine to afford one-dimensional coordination polymers: synthesis and scanning probe microscopy characterization. *Inorg. Chem.* **45**, 7642-7650 (2006).
17 Firefly 7.1 (http://classic.chem.msu.su/gran/firefly/index.html).
18 Schmidt, M. W. *et al.* General atomic and molecular electronic structure system. *J. Comput. Chem.* **14**, 1347-1363 (1993).
19 Stevens, W. J., Basch, H. & Krauss, M. Compact effective potentials and efficient shared-exponent basis sets for the first- and second-row atoms. *J. Chem. Phys.* **81**, 6026-6033 (1984).
20 Stevens, W. J., Basch, H., Krauss, M. & Jasien, P. J. Relativistic compact effective potentials and efficient, shared-exponent basis sets for the third-, fourth-, and fifth-row atoms. *Can. J. Chem* **70**, 612-630 (1992).
21 Battistoni, C., Mattogno, G., Cariati, F., Naldini, L. & Sgamellotti, A. XPS photoelectron spectra of cluster compounds of gold *Inorganica. Chimica. Acta.* **24**, 207-210 (1977).
22 Moulder, J. F. & Chastain, J. *Handbook of x-ray photoelectron spectroscopy: A reference book of standard spectra for identification and interpretation of XPS data.* (Physical Electronics Division, Perkin-Elmer Corporation, 1992).
23 Bourg, M.-C., Badia, A. & Lennox, R. B. Gold-Sulfur Bonding in 2D and 3D Self-Assembled Monolayers: XPS Characterization. *J. Phys. Chem. B.* **104**, 6562-6567 (2000).
24 McNeillie, A., Brown, D. H., Smith, W. E., Gibson, M. & Watson, L. X-ray photoelectron spectra of some gold compounds. *J. Chem. Soc., Dalton Trans.*, 767-770 (1980).
25 Kitagawa, H., Kojima, N. & Nakajima, T. Studies of mixed-valence states in three-dimensional halogen-bridged gold compounds, Cs2AuIAuIIIX6, (X = Cl, Br or I). Part 2. X-Ray photoelectron spectroscopic study. *J. Chem. Soc., Dalton Trans.*, 3121-3125 (1991).
26 Best, S. A. *et al.* X-ray photoelectron spectra of inorganic molecules. 18. Observations on sulfur 2p binding energies in transition metal complexes of sulfur-containing ligands. *Inorg. Chem.* **16**, 1976-1979 (1977).
27 Walton, R. A. The x-ray photoelectron spectra of metal complexes of sulfur-containing ligands: Sulfur 2p binding energies. *Coord. Chem. Rev.* **31**, 183-220 (1980).
28 Castner, D. G., Hinds, K. & Grainger, D. W. X-ray photoelectron spectroscopy sulfur 2p study of organic thiol and disulfide binding interactions with gold surfaces. *Langmuir* **12**, 5083-5086 (1996).
29 Lindberg, B. J. *et al.* Molecular spectroscopy by means of ESCA II. Sulfur compounds. Correlation of electron binding energy with structure. *Physica. Scripta.* **1**, 286-298 (1970).
30 Manceau, M. *et al.* Further insights into the photodegradation of poly(3-hexylthiophene) by means of X-ray photoelectron spectroscopy. *Thin Solid Films* **518**, 7113-7118 (2010).
31 Almukhlifi, H. A. & Burns, R. C. Gold nanoparticles on metal oxide surfaces derived from n-alkanethiolate-stabilized gold nanoparticles; investigations of the adsorption mechanism and sulfate formation during subsequent thermolysis. *Appl. Catal. A Gen* **502**, 174-187 (2015).
32 Chilkoti, A. & Ratner, B. D. X-ray photoelectron spectroscopy of iodine-doped nonconjugated polymers. *Chem. Mater.* **5**, 786-792 (1993).
33 Salaneck, W. R. *et al.* Photoelectron-spectroscopy of iodine-doped polyacetylene. *J. Chem. Phys.* **72**, 3674-3678 (1980).
34 Hsu, S. L., Signorelli, A. J., Pez, G. P. & Baughman, R. H. Highly conducting iodine derivatives of polyacetylene: Raman, XPS and X-ray diffraction studies. *J. Chem. Phys.* **69**, 106-111 (1978).
35 Polzonetti, G., Faruffini, V., Furlani, A. & Russo, M. V. X-ray photoelectron-spectroscopy of iodine-doped polyethynylferrocene. *Synth. Met.* **25**, 375-384 (1988).
36 Polzonetti, G., Furlani, A., Russo, M. V., Camus, A. M. & Marsich, N. Evidence of reacting iodine species at the surface of a polyphenylacetylene film investigated by XPS. *J. Electron. Spectrosc. Relat. Phenom.* **52**, 581-588 (1990).
37 Moulay, S. Molecular iodine/polymer complexes. *J. Polym. Eng.* **33**, 389-443 (2013).
38 Briggs, D. *Surface analysis of polymers by XPS and static SIMS.* (Cambridge University Press, 1998).

39 Sam, S. S. *et al.* Peptide immobilisation on porous silicon surface for metal ions detection. *Nanoscale Res. Lett.* **6** (2011).
40 Kondo, M., Mates, T. E., Fischer, D. A., Wudl, F. & Kramer, E. J. Bonding structure of phenylacetylene on hydrogen-terminated Si(111) and Si(100): surface photoelectron spectroscopy analysis and Ab initio calculations. *Langmuir* **26**, 17000-17012 (2010).
41 Seifert, S., Gavrila, G. N., Zahn, D. R. T. & Braun, W. The molecular orientation of DNA bases on H-passivated Si(111) surfaces investigated by means of near edge X-ray absorption fine structure spectrscopy. *Surf. Sci.* **601**, 2291-2296 (2007).
42 Saprigin, A. V., Thomas, C. W., Dulcey, C. S., Patterson, C. H. & Spector, M. S. Spectroscopic quantification of covalently immobilized oligonucleotides. *Surf. Interface Anal.* **37**, 24-32 (2005).
43 Yue, J. & Epstein, A. J. XPS study of self-doped conducting polyaniline and parent systems. *Macromolecules* **24**, 4441-4445 (1991).
44 Terrill, R. H., Hutchison, J. E. & Murray, R. W. Solid state electron-hopping transport and frozen concentration gradients in a mixed valent viologen-tetraethylene oxide copolymer. *J. Phys. Chem. B* **101**, 1535-1542 (1997).
45 Jernigan, J. C. & Murray, R. W. CONSEQUENCES OF RESTRICTED ION MOBILITY IN ELECTRON-TRANSPORT THROUGH FILMS OF A POLYMERIC OSMIUM POLYPYRIDINE COMPLEX. *J. Phys. Chem.* **91**, 2030-2032 (1987).
46 Watson, S. M. D. *et al.* Synthesis, Characterisation and Electrical Properties of Supramolecular DNA-Templated Polymer Nanowires of 2,5-(Bis-2-thienyl)-pyrrole. *Chem. Eur. J.* **18**, 12008-12019 (2012).
47 Trnkova, L., Studnickova, M. & Palecek, E. ELECTROCHEMICAL-BEHAVIOR OF GUANINE AND ITS DERIVATIVES .1. FAST CYCLIC VOLTAMMETRY OF GUANOSINE AND CALF THYMUS DNA. *Bioelectrochem. Bioenerg.* **7**, 643-658 (1980).
